# Special focus on the ribosome life cycle

**DOI:** 10.1080/15476286.2023.2221946

**Published:** 2023-06-13

**Authors:** Marlene Oeffinger

**Affiliations:** Institut de Recherches Cliniques de Montréal, Montréal, Québec, Canada; Département de Biochimie, Faculté de Médecine, Université de Montréal, Montréal, QC, Canada; Faculty of Medicine, Division of Experimental Medicine, McGill University, Montréal, QC, Canada

In all cells and tissues, protein production is carried out by a ubiquitous machine – the ribosome, a three Mega Dalton macromolecular complex that is essential across all domains of life. It was 65 years ago, in 1958, that the ribosome as protein production machinery was first named by Richard Roberts [1–3]. Five years later, in January 1963, Jonathan Warner, Paul Knopf and Alex Rich published the first paper on the characterization of polyribosomes, or polysomes, from rabbit reticulocyte lysate [4]. The very same year, work by Alfred Gierer in Tubingen, also using reticulocytes [5] and Hans Noll in Pittsburgh, using rat liver [6] independently suggested that protein synthesis was conducted on structures with multiple ribosomes scanning a single mRNA, and in 1964, description of the A(cceptor) and P(eptidyl) sites (the E, or exit, site was only identified by Knut Nierhaus in 1981 [7]) and mRNA and tRNA, followed [8–14]. In the span of a little over a year and a half, views of protein synthesis evolved from a vague idea of an interaction between mRNA, tRNA and ribosomes to a basic model that guides ongoing research into mechanisms of translation regulation to this day: ribosomes move from one end of an mRNA to another, and each has a site for a tRNA attached to the growing polypeptide and a site for the incoming tRNA attached to an amino acid.

It also became clear very early on that the ribosome itself is composed of both RNA and proteins and investigation into its composition and biogenesis began in the 1950s and 60s, in bacteria and eukaryotes, respectively [3]. In 1962, Klaus Scherrer, Harriet Latham, and James E. Darnell demonstrated the existence of precursor ribosomal RNAs (rRNAs) in HeLa cells [15], while R. J. Britten, B. J. McCarthy, and Richard Roberts suggested that ribosomes are formed in a stepwise manner [16,17]. The nucleolus was identified as the site of ribosome biogenesis only 2 years later [18], the same year as rRNA base methylation [19] and both rRNA and ‘nascent’ ribosomes of different molecular mass were isolated from human nuclei for the first time in 1967 [15,19,20].

It was in 1972 that a study by Kumar & Warner on nuclear *vs* cytoplasmic ribosomes in human cells revealed a considerable difference in protein content [21]. At the time, these ‘extra proteins’ within nuclear ribosomes were – in retrospect, correctly – hypothesized to function in the processing of rRNA precursors, and while ribosomes were moved to the cytoplasm, these ‘extra proteins’ stayed behind and were surmised to be re-utilized in further rounds of ribosome production [5]. These ‘extra’ proteins are now known to be the many ribosome biogenesis factors that are required to modify, process, and fold the ribosomal RNAs (rRNA) and assemble them with the ribosomal proteins (r-proteins) into mature ribosomes prior to their transport into the cytoplasm, to the site of translation [22,23].

Without doubt, the ribosome has intrigued many scientists in the decades since its initial characterization, either in its function as a protein synthesis machinery or as a complex macromolecular machine that requires a carefully orchestrated pathway and hundreds of proteins to ensure its assembly. Ribosome research has also come of age with the advances in biochemistry and mass spectrometry in the 2000s and the deep sequencing, high-resolution and cryo-electron microscopy approaches of the last decade. These enabled not only the identification and subsequent characterization of many of the ribosome biogenesis factors but also provided insights into many aspects of the ribosome life cycle: from translation regulation, non-canonical translation initiation and localized translation to ribosome heterogeneity, ribosome and pre-ribosome structures across several species, as well as the intricate link between cell stress and ribosome production, and numerous ribosome-linked disease etiologies or ribosomopathies.

Yet despite our growing understanding of ribosome maturation and function over the years, there are many questions that still remain unanswered. The main purpose of this Special Focus on the Ribosome Life cycle is to review some of these questions and discuss recent findings as well as emerging concepts in ribosome biogenesis, translation, and ribosome biology as a whole.

Collectively genetic, biochemical, and structural studies have revealed many different aspects of ribosomes in bacteria and eukaryotes to near-atomic levels and have provided a detailed understanding of what are now considered well-established models for ribosome biogenesis and function both in terms of model organisms and ribosome biology (encompassing biogenesis, composition, and function) as viewed from a ‘classical’ perspective [24]. However, as both functional and compositional diversity of ribosomes have been suggested in recent years, it may become increasingly important to step outside the framework of these models to explore the functional and structural diversity of both ribosome biogenesis and function across the biological diversity as it exists in cells, tissues, and different organisms. In his point-of-view, Ferreira-Cerca discusses how studying the ribosome in the ‘third domain of life’, archaea, as well as in many non-model bacterial and eukaryotic organisms, which have so far been largely neglected, will not only be important to provide an as yet untapped window into the evolution of ribosome biogenesis and function but also to unravel fundamental principles of the molecular adaptation of these central cellular processes [25].

One such example is the mitochondrial ribosome. Initially identified in 1958 and then more thoroughly described in 1974 [26,27], the mitochondrial ribosome originated from a bacterial ancestor and required substantial changes to adapt to its endosymbiotic environment and highly specialized function. In their review, Nadler and colleagues provide a perspective on the recent progress in understanding the process of protein biosynthesis in mitochondria including the mechanistic and physiological details of translation termination and mitochondrial ribosome function [28]. The authors explore how mitochondrial ribosomes are recycled and rescued in the context of limited translation factors or on aberrant mRNAs and draw comparisons to the ancestral bacterial system. Seely and Gagnon, also focus on the mitochondrial ribosome in their review and consider recently gained knowledge about mechanisms and factors involved in ribosome recycling in mitochondria and eubacteria based on X-ray crystallography and cryo-electron microscopy studies of ribosome complexes [29]. In particular, the authors examine how these steps require the concerted and synergistic action of both ribosome recycling and elongation factors.

But it is not only various step of translation and ribosome recycling that necessitate such concerted effort. The production of ribosomes involves the orchestrated function of hundreds of proteins and small nucleolar RNAs (snoRNAs) to process ribosomal RNA and assemble the ribosomal subunits. While the mechanism of how some of these proteins and snoRNAs facilitate rRNA processing or assembly has been established, for some it remains unknown. In their research paper, Vos and Kothe provide mechanistic insights into the cooperative interactions between ribosome maturation factors and snoRNPs to increase their affinity for ribosomal precursor RNA (pre-rRNA) [30]. Using *in vitro* reconstitution, the authors demonstrate how the synergistic interaction between the *Saccharomyces cerevisiae* snR30 H/ACA ribonucleoprotein (RNP) and the ribosome maturation factor Utp23 enhances snR30 base-pairing with the expansion segment 6 (ES6) of the 18S rRNA and indirectly facilitates processing of the pre-rRNA. Concomitant with processing, the rRNA also has to be folded correctly to produce a functional ribosome. While recent cryo-electron microscopy studies have elucidated the order of folding of rRNA subdomains, the mechanism of folding *per se*, however, is less well understood. In their review, Mitterer and Pertschy discuss the current knowledge of rRNA folding in *S. cerevisiae* and other organisms [31], with a focus on how numerous RNA helicases may contribute to the many important folding and unfolding events during ribosome maturation, as misfolding can not only lead to the formation of aberrant ribosomes and subsequent pre-rRNA degradation but also give potential rise to defective ribosomes that may nevertheless participate in translation.

Under certain conditions such defective ribosomes may cause ribosome collisions, which have been suggested to serve as a central signal for translational stress and can trigger different stress responses. In their point-of-view, De and Mühlemann discuss the intricate mutual connections between ribosome collisions, stress response pathways and mRNA surveillance [32]. In particular, the authors provide insight into the role of the E3-ligase ZNF598, a central factor connecting the step of sensing collided ribosomes with that of degradation of the nascent polypeptides as well as the dissociation of the stalled ribosomes from mRNA and subsequent mRNA degradation *via* no-go or non-stop decay. De and Mühlemann also demonstrate that ZNF598 is dispensable for nonsense-mediated mRNA decay (NMD) and consequently argue against a mechanism of stable ribosome stalling at termination codons as an NMD-triggering signal.

Defects in ribosome biogenesis have also been implicated in a number of diseases and are primarily caused by mutations in ribosome maturation factors. These are mostly congenital and have been termed ribosomopathies [33,34]. In their review, McFadden and Baserga provide insights into the consequences of abnormal functions of the C/D box U8 snoRNA [35]. U8 snoRNA, encoded by the SNORD118 gene, is an atypical C/D box snoRNA insofar as it promotes rRNA cleavage rather than 2’-O-methylation and is unique to vertebrates. The authors detail how U8 snoRNA function in ribosome biogenesis is intricately linked to the preservation of brain function in humans as its dysregulation due to a single nucleotide polymorphism (SNP) in the SNORD118 gene is causative for Labrune syndrome, a neurodegenerative disease whose hallmarks are leukoencephalopathy, brain calcifications, and cyst formation.

The connection between ribosome biogenesis and disease etiology has also given rise to enquiries into so-called ‘rogue’ ribosomes – ribosomes that may be prone to translate pathology-correlated proteins [36]—which, in turn, drew attention to the question of whether ribosomes are indeed the monolithic structures they have been considered to be for the past decades. While their definition is still a highly debated [37,38], substantial evidence now exists for ribosomal heterogeneity both at intracellular and intercellular levels in different organisms [39]. Whether this variation represents a functional ribosomal specialization or rather a tolerated margin of error in cells that is below a pathological threshold is an ongoing key question in translation. Joo and colleagues discuss in their review the current understanding of ribosome heterogeneity highlighting the potential roles of specialized ribosomes in translation [40]. As the authors describe, ribosome heterogeneity may be reflected in ribosomal protein (r-protein) composition and r-protein paralogs or -like factors; modifications of rRNAs or r-proteins; or the binding of ribosome-associated proteins. However, as Trahan and Oeffinger highlight in their point-of-view, ribosomal RNA can equally be a source of ribosome heterogeneity and differential translation regulation [41]. In particular, the authors consider ribosome heterogeneity from an evolutionary standpoint, with a focus on the nucleic acid level, and overall argue for a reframing of ribosome ‘heterogeneity’ as an adaptive and dynamic process of plasticity.

One of the many still remaining challenges in the field will be to determine and confirm a potential differential function of heterogeneous ribosomes on select mRNAs. But new approaches to study ribosomes are continuously emerging. In their technical paper, Gurzeler and colleagues present a protocol to produce translation-competent lysates from human cells or tissues [42]. In their approach, the authors use an optimized lysate preparation based on dual centrifugation that allows for detergent-free cell lysis undercontrolled mechanical forces and yields cytoplasm-enriched extracts from human cells that efficiently translate mRNAs in a cap-dependent as well as in an IRES-mediated way.

The collection of articles in this special issue of RNA Biology presents some of the current knowledge on the biology of the ribosome and its life cycle. Altogether, they demonstrate that even after more than six decades of research, there is still much to learn about the various facets of the ribosome – its biogenesis, composition, potentially heterogenous function and translation targets, and, of course, how these are implicated in the etiology of various pathologies.
